# In the Shadow of Smoke and Silence: Understanding Cancer Awareness and Healthcare-Seeking Behavior in Louisiana’s Cancer Alley

**DOI:** 10.3390/healthcare14193138

**Published:** 2026-09-22

**Authors:** Moumita Roy, Jinx Coleman Broussard

**Affiliations:** Manship School of Mass Communication, Louisiana State University, Baton Rouge, LA 70803, USA; jinxy@lsu.edu

**Keywords:** environmental health disparities, medical mistrust, health information seeking, preventive healthcare, healthcare access

## Abstract

**Highlights:**

**What are the main findings?**

- Resident participants perceived a high risk of pollution-related cancer, yet awareness of cancer services, preventive screenings, and available healthcare programs within 100 miles remained limited.
- Mistrust, financial constraints, transportation barriers, distance from specialists, and insufficient health communication were described as factors shaping delayed and largely reactive healthcare-seeking behavior.

**What are the implications of the main findings?**

- Conventional, one-way health communication is inadequate in communities shaped by environmental injustice and institutional mistrust; healthcare organizations must adopt sustained, culturally responsive, two-way outreach through trusted community networks.
- Improving cancer outcomes requires more than increasing awareness: interventions must address transportation, affordability, specialist access, preventive screening, and institutional accountability to reduce delayed care in industrially polluted communities.

**Abstract:**

**Background/Objectives**: Louisiana’s industrial corridor, commonly known as “Cancer Alley,” has been extensively documented as a site of environmental and health inequity. Less is known about how residents understand cancer risk, identify available cancer care, and make decisions about seeking healthcare. This study examined cancer awareness, healthcare-seeking experiences, and communication-related barriers among residents of Cancer Alley. **Methods**: This exploratory qualitative case study used nine semi-structured interviews: seven with Cancer Alley residents, one with a journalist familiar with the region, and one with a healthcare communication professional. Guided by the Comprehensive Model of Information Seeking (CMIS), the Health Belief Model (HBM), and the Theory of Health-Seeking Behavior (THSB), the study examined awareness of cancer services, perceived susceptibility, barriers to care, and communication experiences. **Results**: Resident participants described uneven knowledge of nearby cancer facilities and limited awareness of specific services and preventive screening opportunities. Financial constraints, transportation burdens, distance from specialists, mistrust, and personal or familial cancer experiences also shaped healthcare-seeking decisions. A notable discrepancy emerged between residents’ reports of limited outreach and the healthcare communication professional’s description of existing outreach and free screening programs, suggesting a gap between service availability and community awareness. Participants frequently perceived themselves as vulnerable to cancer but described largely reactive rather than preventive healthcare-seeking. **Conclusions**: These findings do not establish population-level prevalence or causal relationships; rather, they identify communication and access mechanisms that warrant further investigation. Sustained, community-centered, two-way communication about available services may help strengthen awareness and support preventive healthcare engagement in environmentally burdened Louisiana communities.

## 1. Introduction

Cancer Alley, an 85-mile stretch along the Mississippi River between New Orleans and Baton Rouge, hosts about 200 petroleum refining and chemical manufacturing facilities, making it one of the most industrialized regions in the U.S. However, “Cancer Alley” does not exist on maps. Pollutants the chemical plants emit daily prompted that designation because of the high prevalence of cancer among locals. The coexistence of residents with fossil fuel and petrochemical industries exposes them to extreme pollution, making parts of Cancer Alley the highest-risk areas for cancer from industrial air pollution in the U.S. [1]. Health risks and disparities extend beyond cancer to respiratory issues and maternal and newborn health complications [1], particularly in Black and African American communities in Cancer Alley [2]. The state’s high poverty rate exacerbates these disparities, leading to disproportionately high mortality rates for all cancer types [3]. Environmental-level exposures, lack of access to healthcare services due to geographical constraints, transportation issues, existing beliefs, and a lack of awareness about available services [4] are all barriers that contribute to the increasing rate of cancer patients [4,5]. Addressing these disparities is imperative to ensure equitable access to quality cancer care and improve health outcomes for underserved populations in Louisiana.

Grounded in three health behavioral theories—the comprehensive model of information seeking, the health belief model, and the theory of health-seeking behavior—this research addresses a critical gap in understanding healthcare-seeking behaviors in Cancer Alley, where severe health crises persist despite limited studies on residents’ knowledge of and access to cancer care. Investigating environmental and healthcare inequities in this polluted landscape, the study examines residents’ awareness of cancer treatment services within a 100-mile radius and how this impacts their care-seeking decisions. It identifies barriers to timely and adequate healthcare while exploring the interplay of environmental racism, healthcare access, and cancer outcomes. Perspectives from residents, environmental justice advocates, healthcare communication professionals, and journalists provide a comprehensive view of these challenges. Grounded in three health behavioral theories, the comprehensive model of information seeking, the health belief model, and the theory of health-seeking behavior, the study also offers participant-driven recommendations on effective communication strategies to better reach marginalized Black communities in Cancer Alley.

Pezzullo (2003) explored racial and ethnic health disparities and environmental racism in Baton Rouge, addressing concerns similar to this study about Cancer Alley’s hazardous landscape from a communication perspective [6]. Her study remains the only communication-focused research on this issue, emphasizing the need to merge nature and culture to raise awareness and inspire action for environmental justice. She introduced “toxic tours” as cultural spectacles that politicize memory, unite activists, and restore communities. Despite its reprint in 2010, no scholar has expanded on her work to examine how communication informs Cancer Alley residents about environmental health risks or influences their cancer-related information-seeking behaviors. This absence may explain why racial and ethnic health disparities persist in the Southern U.S.

## 2. Literature Review

### 2.1. Environmental/Structural Racism

Once home to sugar and cotton plantations, the Louisiana Bayou is now one of the most polluted areas in the U.S. due to petrochemical manufacturing [7]. Before becoming “Cancer Alley,” this region was known as “Plantation Country,” where enslaved Africans were forced to work. Pollution surged in the 1980s, bringing severe health issues [8].

Environmental racism and geographic disparities in Louisiana’s Cancer Alley heighten residents’ cancer risk [9]. Despite a 1987 Washington Post exposé on hazardous pollution [10], efforts to curb toxic emissions remain inadequate. This petrochemical corridor has polluted water and air, exposing its predominantly African American residents to cancer, respiratory diseases, and other health issues [11]. According to Terrell et al. [5], socioeconomic factors exacerbate health risks in this area, as many residents lack financial resources for medical care or legal action. Human Rights Watch [1] found 12 sites exceeding federal pollution limits and documented fossil fuel plants near schools, homes, and playgrounds in nine Cancer Alley parishes such as Ascension, Iberville, Orleans, St. Charles, St. James, St. John the Baptist, Jefferson, East Baton Rouge, and West Baton Rouge, forcing residents to endure constant toxic exposure. Research also highlights systemic environmental inequality. Mohai et al. (2009) found low-income, less-educated populations live closer to polluting sites, reinforcing how economic and racial inequities dictate exposure to harmful emissions [12].

### 2.2. Environmental and Health Issues Prevalent in the Area

Human Rights Watch [1] reported that Cancer Alley residents face extreme pollution, increasing cancer, respiratory, and maternal health risks [1]. Terrell et al. (2024) found 27 in 100 newborns have low birthweight, over triple the national average, while preterm births (25.3%) are nearly 2.5 times the U.S. rate [5]. Asthma and lung disease are also widespread. St. John Parish’s asthma rates for children are 2.5 times the state and national averages, with hospitalization rates equally high [13]. Lung cancer is common among those exposed to industrial pollutants [14], and workplace chemical exposure is a known risk factor [15]. Despite approximately 20,000 people still residing in Cancer Alley [8], HRW [1] criticized Louisiana’s environmental agencies for failing to act. The EPA identified Cancer Alley as a toxic hotspot, with cancer risks 700 times the national average in some parishes [16]. Annually, 85 cancer cases in Louisiana are linked to air pollution [5].

### 2.3. Barriers to Cancer Healthcare

One of the main barriers to cancer healthcare in Cancer Alley is transportation challenges. A study using data from the National Health Interview Survey (1997–2017) found that 5.8 million people in the United States (1.8%) postponed medical care due to a lack of transportation [17]. Cochran et al. (2022) reached the same conclusion in their study, which revealed that 35.3% of 114 respondents and 18.3% of 59 respondents delayed or missed medical appointments or treatments in the past year due to transportation barriers, while 16.4% of 53 respondents reported arriving late [18]. Another factor contributing to healthcare disparities is access. Levit et al. (2020) highlight that long travel times, limited access to medical and oncology professionals, and low clinical trial enrollment negatively affect patient care and outcomes [19]. Poor financial conditions and limited healthcare services are also significant barriers for cancer patients, as rising healthcare costs make it difficult for many patients to access necessary treatment. Mathur et al. (2015) found that 18.1% of individuals faced financial barriers to healthcare services, while 12.9% struggled with medication costs [20]. Oncology providers are concerned about these economic obstacles hindering access to innovative treatments [21], which can result in “financial toxicity” for low-income patients in Louisiana. Carrera et al. (2022) note that financial circumstances, including health insurance coverage, impact care-seeking decisions [21]. Lack of insurance or high deductibles can prevent access to cancer screenings or treatments, indirectly leading to higher cancer mortality.

Medical mistrust remains a major obstacle to building strong patient–clinician relationships. Mistrust often stems from experiences of discrimination [22]. A 2020 poll revealed that 7 out of 10 Black Americans believe the healthcare system treats them unfairly, with 55% expressing outright distrust [23]. Such distrust is deeply rooted in historical injustices, including the 1932 Tuskegee Syphilis Experiment [24], 20th-century forced sterilizations of African American women [25], and other unethical medical practices. For many vulnerable individuals, especially those who are doubly marginalized, these past and present experiences of mistreatment continue to fuel a profound distrust in the healthcare system [25].

Racial disparities in healthcare remain a serious social issue, especially in the Southern U.S., where environmental and health challenges are widespread. Yet, little scholarly attention has been given to structural racism and healthcare barriers in Cancer Alley, leading to the central research question (RQ1).

**RQ1.**  *To what extent are residents of Cancer Alley aware of cancer care within a 100-mile radius of their homes, and how does this, along with other influences, impact their care-seeking behavior?*

## 3. Theoretical Background

### 3.1. Comprehensive Model of Information Seeking (CMIS)

CMIS predicts health information-seeking behaviors based on health beliefs and medium-related characteristics [26]. It suggests that health-related factors influence information carrier factors, which in turn affect information-seeking behaviors [27]. Four elements influence information seeking: demographic factors (age, gender, education, ethnicity, and socioeconomic status), direct experiences (health conditions and social networks), salience (gaps or issues driving information need), and beliefs (shaping perceptions) [28,29,30]. These factors affect how individuals seek information about health, yet it remains unclear how these influences impact residents of Cancer Alley in seeking cancer care, potentially contributing to knowledge and access gaps. As such, research question two is posed:

**RQ2.**  *What are the key factors that influence the decision-making process of residents in Cancer Alley regarding seeking cancer care and other medical treatment?*

### 3.2. Health Belief Model (HBM)

According to HBM, health behavior is influenced by beliefs regarding disease severity, personal vulnerability, perceived benefits, and barriers to action [31]. The four key constructs of HBM include perceived susceptibility, perceived severity, perceived benefits, and perceived barriers, with the first two reflecting threat dimensions and the latter two representing net benefits. Additionally, “cues to action” and “self-efficacy” drive behavior change [32,33].

Clinicians can use the model to understand how individuals in the precontemplation stage may need support to see the positive outcomes of behavior changes [34]. This model is crucial in understanding how Cancer Alley residents perceive health risks and how such perceptions might affect their healthcare-seeking behavior. Therefore, the research question is:

**RQ3.**  *How do residents of Cancer Alley perceive their susceptibility to cancer, and how does this perception influence their healthcare-seeking behavior?*

### 3.3. Theory of Health-Seeking Behavior (THSB)

THSB suggests that individuals engage in health-seeking actions to treat or cope with symptoms, as they perceive themselves to have a health problem [35]. Chrisman [36] described health-seeking behavior as a complex process, starting with symptom recognition, followed by interpretation, cause attribution, and decisions on treatment options. This process is influenced by sociocultural factors and socioeconomic status, including exposure to the biomedical system [36].

The model highlights that health-seeking behavior is shaped by past experiences and can vary based on available treatment options. Understanding the health-seeking process is vital, particularly in communities like Cancer Alley, where exposure to healthcare options plays a crucial role in treatment decisions [35,36]. Thus, research question four is:

**RQ4.**  *How does awareness and exposure to the healthcare system affect the treatment-seeking behavior of Cancer Alley residents?*

## 4. Methodology

### 4.1. Researcher Positionality and Reflexivity

The first author, who conducted the interviews and primary analysis, is an Asian female graduate student in the Southern United States whose interest in this topic was shaped partly by recent family experiences with cancer. Although she has not personally experienced cancer, witnessing its effects within her family contributed to her interest in how individuals and families understand cancer risk, healthcare information, and treatment decisions. Her research has focused on health communication and health-related decision-making.

The first author did not share the racial identity or long-term community membership of most resident participants. This partial outsider position is relevant because the study addresses environmental injustice, healthcare mistrust, and experiences within predominantly Black communities. Differences in racial, cultural, and community background may have influenced rapport, what participants chose to disclose, and how their accounts were interpreted. The second author is an African American professor and scholar with more than two decades of academic and professional experience in mass communication. Her research includes the Black press, representations of racial and ethnic minorities, media history, the civil rights movement, and public relations. Her scholarly background provided contextual sensitivity to issues of race, representation, and institutional trust relevant to this study, while the first author conducted the interviews and primary analysis. Accordingly, researcher positionality was treated as part of the interpretive context rather than as evidence of neutrality. Qualitative reporting guidelines emphasize that researcher characteristics and interviewer-participant relationships should be made explicit because they may shape data collection and interpretation [37]. The interviewer had no established relationship with participants before recruitment.

### 4.2. Study Design and Sampling

This study employed an exploratory qualitative case-study design, with Louisiana’s Cancer Alley serving as a geographically and socially bounded case. Case-study research is appropriate for examining complex phenomena within their real-life contexts and can incorporate qualitative interviews as a primary source of evidence [38]. Within this bounded case, semi-structured, in-depth interviews were used to examine participants’ experiences and perceptions of cancer risk, healthcare awareness, and care-seeking.

Participants were recruited through social advocacy organizations, social media, direct outreach, and snowball sampling. Due to residents’ mistrust and limited research access in the region, snowball sampling was used to reach participants [39,40]. All participants (see Table 1) gave informed consent before interviews, which were conducted via Zoom or phone, depending on participant access and technical circumstances, and lasted between 30 and 60 min. To protect confidentiality, researchers replaced names with pseudonyms in transcripts, and participants had the option to use coded identifiers (e.g., 001, 002) on Zoom before recording began.

The study included nine participants. Seven were residents of Cancer Alley and constituted the primary experiential sample for questions concerning awareness, risk perception, and healthcare-seeking. Two additional participants—a journalist familiar with Cancer Alley and a healthcare communication professional were included as contextual informants because their professional experiences provided perspectives on public information, community outreach, and healthcare communication. Their accounts were used to contextualize and compare with resident experiences rather than to represent resident healthcare-seeking behavior.

Given the exploratory design and small, purposively recruited sample, the study does not claim statistical representativeness. Data saturation was not prospectively documented; therefore, saturation is not claimed retrospectively. Qualitative sample adequacy depends not only on participant numbers but also on the study aim, sample specificity, use of theory, quality of dialogue, and analytic strategy, a principle described by Malterud et al. (2016) as “information power.” At the same time, additional interviews across a broader range of Cancer Alley communities could reveal experiences or themes not represented in the current sample [41]. Accordingly, the findings should be interpreted as an in-depth exploration of these participants’ experiences rather than as representative of all Cancer Alley residents.

To interpret the study’s findings, participants’ pseudonyms and abbreviations for race and gender (e.g., Black Female = BF, Black Male = BM, White Female = WF, White Male = WM, Hispanic or Latino Female = HLF, and Asian Female = AF) are used. This coding allows for quick referencing and ensures an accurate understanding, considering the study’s ethnographic and cultural elements.

### 4.3. Data Analysis

Interview data were analyzed using a primarily deductive, semantic thematic approach within the bounded Cancer Alley case. The initial coding process was informed by the research questions, prior literature, and the three theoretical frameworks and therefore included sensitizing concepts such as healthcare seeking, perceived susceptibility, mistrust, awareness, accessibility, and communication. A deductive orientation does not preclude the identification of relationships or tensions that extend beyond predefined codes; thematic analysis requires moving from individual coded segments toward broader patterns of shared meaning across the dataset [42,43].

Following transcription and repeated familiarization with the interviews, relevant data segments were coded and compared across participants. Codes were then reviewed for relationships, contrasts, and recurring patterns rather than being retained as direct equivalents of the research questions. Although the initial coding was guided by the research questions and theoretical frameworks, the analysis also examined relationships across predefined domains and allowed cross-cutting patterns to be identified that had not been specified as separate a priori categories. These emergent relationships informed the final thematic interpretation rather than being predetermined by the research questions. This process produced four broader themes: (1) knowing healthcare facilities does not necessarily mean knowing available cancer services; (2) willingness to seek quality care is constrained by financial, transportation, specialist-access, and trust barriers; (3) high perceived cancer susceptibility can coexist with reactive rather than preventive healthcare-seeking; and (4) a gap can exist between healthcare outreach being offered and residents’ awareness of that outreach. The last two themes were especially important because they reflected tensions across participants’ accounts rather than simply reproducing the concepts specified in the interview questions.

The three theoretical frameworks were applied during interpretation rather than treated as four separate coding templates. The Comprehensive Model of Information Seeking (CMIS) informed interpretation of information needs, prior experience, and perceptions of information sources; the Health Belief Model (HBM) informed interpretation of perceived susceptibility and barriers; and the Theory of Health-Seeking Behavior (THSB) informed interpretation of how symptom recognition, prior experiences, and healthcare-system exposure shaped care-seeking. This integrated approach allowed the analysis to examine how information, perceived risk, structural barriers, and treatment decisions interacted within the same participant accounts. The first author conducted the primary coding and thematic analysis. The second author reviewed the emerging themes and interpretation of the findings and provided feedback to strengthen the consistency and credibility of the analysis.

## 5. Results

The nine-person sample included seven Cancer Alley residents and two contextual professional participants. Among the seven residents, five identified as Black or African American, one as White, and one as Hispanic or Latino. The professional participants included one journalist and one healthcare communication professional; their accounts are distinguished from residents’ experiences throughout the analysis. Participants spanned five age groups, with most aged 65+. Five residents were from St. James Parish, two from Iberville, and both professionals were from East Baton Rouge. Educational backgrounds varied: two had a high school diploma or GED, five held bachelor’s degrees, and two had postgraduate degrees, though education level showed no clear link to healthcare-seeking behavior. Three residents working in chemical plants had cancer. Income disparities were evident, with plant workers in St. James earning less than those in Iberville, while higher education correlated with higher income in other sectors. Data from nine in-depth interviews (6 female, 3 male) addressed the research questions, with key findings summarized in Table 2.

Five residents had lived in Cancer Alley their entire lives, recalling cleaner air and water in childhood. Some remained due to proximity to Baton Rouge and New Orleans, while others stayed for family roots. According to Latoya (BF), “St. James is home to me, and I have been here all my life. And I would not want to be any other place in this world, because this is where my roots are.” Tyrone (BM), on the other hand, says, “I pledged myself to stay in this area to take care of the people who took care of me, and that is my mom and my dad. I am living in the family house right now, but that is the reason why I am still here.”

All seven residents were mostly aware that pollution was causing health issues such as cancer, low birth weight, and respiratory illnesses. Six of them knew that the area they live in is called “Cancer Alley” and expressed distress over it. Lucia (HLF) said, “It makes me feel less confident about living in that area because it, you know, makes me think that I have a higher risk for cancer than somebody else.” All seven residents also agreed that the plant has a major influence on their health and causes death through the pollution it emits. Melisa (BF) argued that there were no plants in this area when she grew up, and it was in the late ‘70s when the plants started to come in. She also said, “The impact of these chemical companies is that it’s causing us to be sick.”

Participants expressed differing views about the economic benefits associated with petrochemical industries. Several emphasized that employment and industrial revenue did not compensate for perceived health and environmental harms, while others acknowledged employment, lower taxes, or employer-provided healthcare as economic benefits. According to Latoya (BF), “there is no financial gain for the residents; the financial gains are for the industries. The industries are polluting us, and they are getting profits. We are getting sick and fighting for our lives, not them.” Conversely, Lucia (HLF) said, “Financially, I think they’re in a better situation because our taxes are lower, and a lot of us work for the plants. So, financially, they were affected positively.” April (WF) said that “Although I should mention that a lot of people are employed at these companies, and they have top-notch healthcare if they are employed there, I don’t think it necessarily makes up for it. Overall, I think it’s a negative effect.” Although April (WF) acknowledges the employment and healthcare benefits from industries, she, unlike Lucia (HLF), sees the overall impact as negative, aligning more with Latoya’s view on the harmful effects.

### 5.1. Knowing Where Care Exists Is Not the Same as Knowing What Care Is Available

Beyond the predefined domains, comparison across participant accounts revealed three cross-cutting patterns: a service-awareness gap, a tension between willingness to travel and practical access barriers, and the coexistence of high perceived cancer risk with reactive care-seeking. This study revealed mixed awareness of cancer care within a 100-mile radius of Cancer Alley. Residents’ knowledge varied based on personal experiences and family history of cancer. While some residents were very aware of the healthcare provided by nearby hospitals within or near the parishes, some residents showed limited awareness. For example, April (WF) and Lucia (HLF) were highly aware of the available cancer care within Cancer Alley and mentioned a couple of facilities they are aware of, such as Our Lady of the Lake in Baton Rouge, Mary Bird Perkins Cancer Center in Baton Rouge, and Women’s Hospital. Both April and Lucia live in Iberville, and these healthcare centers are 32.8 miles, 32.5 miles, and 37.4 miles away, respectively, from Iberville. Both had a family history of cancer, and both learned about these cancer care facilities from their mother’s cancer care journeys. Lucia herself is also a cancer survivor who suffered from breast cancer. Another participant, Marcus (BM), who lives in St. James Parish, also showed awareness and talked about Ochsner in New Orleans (50.2 miles), Baton Rouge Medical (44.1 miles), Mary Bird Perkins Cancer Center in Baton Rouge (36.4 miles), and Thibodaux Regional Health System (36.3 miles). It is important to note that he also has personal experience with cancer and has suffered from it.

In contrast, Jasmine (BF), another St. James resident, demonstrated limited knowledge of nearby cancer care and said, “It would have to be Ochsner in New Orleans. I don’t think our local hospital does any cancer care.” The parish is 50.2 miles from Ochsner. Her awareness of this healthcare provider grew because of her daughter and some family members having to use Ochsner’s services for all their general healthcare needs. Another participant expressed mixed awareness about the services provided by surrounding hospitals, claiming that Terrebonne General Medical Center lacked cancer care facilities, even though it has. St. Elizabeth Hospital is located in Gonzales, Louisiana, approximately 20.1 miles from St. James Parish. St. James Hospital is 7.8 miles from the parish, whereas Terrebonne General Medical Center is 50.7 miles away.

As an important element, the proximity to high-quality cancer care influenced participants’ decisions regarding where to seek treatment. Almost all the participants were ready to travel long distances to have quality cancer care if they knew they had cancer. For example,

Latoya (BF): “I will travel all the way. I will travel because I want to live. So whatever treatment or whatever I must do to get rid of it, I am going to take it.”

Jasmine (BF): “For cancer care specifically, I would definitely be willing to travel. And this might sound insane, but a lot of my family does travel to Texas to receive cancer care because it’s supposed to be the best type of care that you could receive in proximity to us.”

According to the findings, factors such as financial considerations, convenience, and family experiences impacted participants’ care-seeking behavior. Tyrone (BM) said, “As long as the trip that I take is taken care of financially, I don’t have a problem going anywhere.” This suggests that although people are willing to travel long distances, financial constraints play a great role in decision-making. Previous experience and family history with cancer is other important factors that influence cancer care-seeking decisions. When asked, participants offered the following responses:

April (WF): “If I had cancer or was worried about cancer, I would drive as far as I had to. Luckily, Our Lady of the Lake in Baton Rouge is really good. I saw them myself to screen for breast cancer, and my mom also went there.”

Lucia (HLF): “My mother had breast cancer. And in the course of her getting treated, I looked into the cancer treatment centers in the area.”

Family history and personal experiences with cancer, as shown by April and Lucia, strongly influence their decisions to seek care. Both participants mentioned their experiences with cancer screening and treatment for themselves or family members, indicating that individuals who have witnessed cancer firsthand are more proactive and knowledgeable about available healthcare options. This underlines the role of personal and familial medical history in motivating healthcare-seeking behaviors.

### 5.2. Access to Care Is Shaped by Intersecting Financial, Geographic, and Trust Barriers

This study uncovered several interrelated factors, such as distrust in the healthcare system, financial limitations, cost of transportation, cost of medical treatment and medications, absence of cancer specialists in nearby hospitals, proximity to healthcare, and accessibility. Individually and collectively, these factors affect people’s overall healthcare-seeking actions. Another key outcome is that these factors are frequently influenced by trust, individual circumstances, generational differences, and personal experiences with the healthcare system.

Trust emerged as one of the factors influencing care-seeking among the interviewed residents. Mistrust was particularly evident in the accounts of Tyrone and Jasmine, who described skepticism or hesitation toward healthcare providers. Tyrone (BM) thinks that “A lot of people do not believe in certain medicines or certain treatments, and so they won’t do it.” Other participants share similar concerns and observations:

Jasmine (BF): “The older community definitely has a lot of distrust towards going to the doctor. I know that they are usually very hesitant about things like surgeries, be they minor or major, even routine checkups. They do not feel comfortable enough to go to the doctor to take care of themselves. It takes a lot of persuasion, but the younger generation is realizing the importance of maintaining our health and becoming more aware of it.”

Tyrone (BM): “The doctors are with the hospital, the hospital is with the doctor, and they are both with pharmaceutical companies. They can cure people quicker. They can cure more people, but they are not doing it because the money is in the treatment. The pharmaceutical companies have the cure for almost every disease that we have. But it is thinking better business if they treat the symptoms. Not the cure.”

Tyrone, a 65+ Black male, expressed deep skepticism toward the healthcare system, believing it to be “an agenda to decrease certain amounts of people in certain ethnic groups,” making it difficult for him to trust any healthcare providers. Jasmine (BF) echoed this sentiment, stating that it is hard to entrust one’s life to someone without full confidence in them. Lucia (HLF), however, expressed greater trust in healthcare and stated that she believed physicians were trustworthy and acted in her best interest. Given the small and uneven racial composition of the sample, these differences are presented as individual participant perspectives rather than evidence of racial-group differences in healthcare trust.

Each of the seven resident participants expressed some degree of mistrust toward government institutions, although their trust in local leaders varied. While at least half trust local leaders, all seven mistrust the government. Tyrone (BM) said, “I think the government may act like they are supporting us in Cancer Alley, but they are not.” Lucia (HLF) noted, “I do not think my neighbors and I trust the government. No, not Louisiana state government at least. There is more trust for local governments because, I guess, it is closer to home.”

Financial hardship and healthcare accessibility also limit residents’ access to cancer care. The high cost of transportation, treatment, and medications remain a significant barrier. Living in rural areas with limited local healthcare forces residents to travel long distances for treatment. Tyrone (BM) stated, “If they do not have transportation or the financial means, they will not go. Who’s going to pay for it?” Jasmine (BF) echoed the concern, emphasizing financial hardship. She said,

“I know how tough it is to obtain the necessary care if you do not have financial resources. And, because this is a rural region, a common topic of discussion is transportation and access to care.”

In response to accessibility, Latoya (BF) highlighted the absence of cancer specialists in their immediate area, necessitating trips to Baton Rouge or New Orleans for specialized care. She said, “We do not have a specialist over here in this area because we live out in a rural area.” April similarly described high-quality physicians as being located farther away. Participants also described traveling up to five to seven hours to Texas to get adequate cancer care. For certain residents like Jasmine (BF), Melisa (BF), Tyrone (BM), and Marcus (BM), their proximity to healthcare is an essential factor that influences their care-seeking behavior. Tyrone (BM) said, “Due to financial constraints, they are unable to travel far from home to receive cancer treatment. The only thing stopping them is that they do not finance transportation.”

### 5.3. High Perceived Cancer Susceptibility Coexists with Reactive Care-Seeking

The study found a strong connection between residents’ perceived cancer risk and their healthcare-seeking behavior. The seven resident participants described heightened cancer vulnerability associated with living near chemical plants and pollution. Personal and community experiences reinforced this perception, with each reporting at least one family member or themselves affected by cancer, with some citing four to six cases. Commonly mentioned cancers included throat, breast, colon, and prostate. Participants also reported other serious conditions, such as chemical-related genetic mutations, asthma, autoimmune hepatitis, Hashimoto’s thyroiditis, and Hashimoto’s encephalopathy:

Latoya (BF): “There are a lot that already have died from cancer. My neighbor on one side had throat cancer. The other one had cancer, I think in the bone or the blood, or whatever; she died. And down this stretch in the 5th district (St. James Parish), we have a lot.”

Tyrone (BM): “I am going to start with my grandfather. My grandfather had one arm, and that’s from working in the plant. My grandmother had breast cancer. I believe she had one of them amputated. My mother did the same thing. My aunt did the same thing. My brother, who worked in chemical industries, developed liver cancer. Working inside those gas and oil tanks damaged his liver, and he died as a result. And three to four other individuals who worked with us died, while two of us already had cancer. I was one of them. I am currently undergoing chemotherapy after being diagnosed with colon cancer.”

As these narratives show, participants have experienced cancer either personally or through close relationships, leading to a heightened sense of vulnerability. The frequency of cancer cases among family, friends, coworkers, and neighbors made residents feel more susceptible. Participants expressed that living in Cancer Alley undermines their confidence in their health and makes them believe they are more likely to develop cancer than others. For example, Marcus (BM) shared that he suffered from throat cancer, which he believes was caused by inhaling the various chemicals in the area. His wife also suffered from breast cancer.

These accounts highlight how direct experiences with cancer among family and friends intensify feelings of vulnerability within the community. Despite awareness of pollution, its link to health issues, and the perceived susceptibility to cancer, this study found minimal proactive healthcare-seeking behavior among participants. Financial constraints, accessibility, and mistrust of the healthcare system hindered their ability to seek medical care. Additionally, skepticism about healthcare influenced their behavior. Many participants with cancer or other complex health conditions only learned of their disease during routine or annual checkups. Despite awareness of the health issues around them, they did not engage in preventive actions such as frequent checkups or screenings. Lucia (HLF) said, “I never really got sick. They found a lump on me, and they had to do surgery to remove it.” Tyrone (BM) said, “I was hurting so much; my nose was bleeding, and I was getting wet. But I never got checked.” Jasmine (BF) explained she will not seek healthcare unless it is something extremely serious, or something that risks her life.

Only one of the seven resident participants described proactively obtaining breast cancer screening, which followed a family history of cancer involving her mother and grandmother. Other participant accounts reflected largely reactive healthcare-seeking, including seeking care after symptoms became serious or learning of health conditions during routine examinations.

### 5.4. The Service-Awareness Gap: Outreach May Exist Without Reaching Residents

This study revealed mixed levels of awareness about available healthcare services among Cancer Alley residents. While two residents (April and Latoya) believed some programs might exist, others expressed a clear lack of information. Despite quality healthcare facilities within 100 miles, Latoya (BF), also an environmental justice advocate, felt cancer screening efforts were poorly communicated: “I am pretty sure they have something. They might screen for something, but I never heard them say they screen for cancer just out of the blue.” In contrast, Romela (AF), a healthcare communication specialist, said, “Our organization (Cancer Center) does a lot of outreaches. And we have a free screening program for cancer screenings.”

A close look at these two responses clearly indicates that there is a gap in awareness, where residents are unsure about the services provided by the healthcare organization. This is where a two-way communication gap arises. Two-way communication works with both the sender and the receiver to acknowledge and understand the message in question and send a response via the same channel [44,45]. Two-way communication emphasizes dialogue and reciprocal exchange rather than one-directional information transmission [45]. This finding is particularly relevant to the CMIS because information seeking depends not only on the existence of information but also on individuals’ perceptions of the usefulness and accessibility of information carriers [26,27]. The finding also suggests that measuring organizational outreach activities alone may overestimate effective community reach: communication cannot be assumed to have occurred merely because information was disseminated.

The study also revealed that little or no health information about different health issues had been circulated in their community in the last six months. Latoya (BF) said, “I don’t think there was any special communication. Not in St. James. If it is, I have not seen it.” April (WF) echoed the same emotion, saying, “What is it? Basically, nothing ever.” While seven residents and one journalist admitted that there is a lack of communication, information, and action for cancer care in this high-risk geographical location, at least three residents mentioned community environmental advocates and advocacy organizations that are working to raise awareness among these vulnerable individuals, such as Rise St. James in the St. James Parish. One even talked about how the healthcare organization is still talking about COVID. Lucia (HLF) said the only thing she had heard about was COVID-19 and the flu and RSV (respiratory syncytial virus). John (WM), a journalist who worked for Cancer Alley News, also emphasized the communication efforts during COVID-19. He said, “I mean, there were definitely efforts during COVID-19 to reach some of those communities and get shots out.” He added, “Recently, I don’t know. Nothing comes to my mind.”

These perceptions suggest that awareness is driven more by personal effort than by systematic outreach. A lack of information about available services and preventive care directly affects treatment-seeking behavior. Building healthcare knowledge requires ongoing communication, especially in communities with deep-rooted mistrust.

## 6. Discussion

Previous research on Cancer Alley linked air pollution to cancer and other health issues [46] but struggled to analyze healthcare-seeking behavior in an environmentally burdened landscape. Given the critical need for medical care in Louisiana’s chemical corridor, this study examined residents’ healthcare awareness and care-seeking behaviors through nine in-depth interviews.

Findings align with prior research identifying Cancer Alley as a major U.S. hotspot for toxic pollutants [16]. Consistent with Terrell et al. (2024) [5], participants’ accounts were situated within a broader context of environmental and social inequities. The interviews additionally identified mistrust in healthcare and government as factors shaping healthcare-seeking behavior. While many residents knew of nearby cancer hospitals, awareness of specific services and preventive care was lacking. Health-seeking behaviors were influenced not only by finances, convenience, and accessibility but also by personal and familial cancer experiences. Financial constraints, proximity to healthcare, lack of specialists, and distrust in the system further shaped behavior. Importantly, the findings revealed cross-cutting tensions that extended beyond the original research-question domains, particularly the gap between available outreach and resident awareness, the contrast between willingness to travel and practical access constraints, and the coexistence of high perceived cancer susceptibility with reactive care-seeking. These results align with the Comprehensive Model of Information Seeking (CMIS), which emphasizes demographic factors and personal experiences in health decisions [27,29]. However, the findings also extend this interpretation by showing that awareness of a healthcare facility does not necessarily mean that residents know what cancer services or preventive programs are available there. In this study, personal and familial cancer experiences appeared to make cancer information more salient for some participants, whereas limited communication about specific services restricted the usefulness of that awareness. Thus, the findings suggest that information seeking is shaped not only by individual experiences and information needs but also by whether relevant healthcare information is accessible and meaningful to residents. Unlike Carrera et al. (2018), who linked financial constraints to insurance coverage, this study found no mention of insurance as a barrier [21]. A key issue was distrust, particularly among some of the Black participants interviewed, with Tyrone’s belief that pharmaceutical companies prioritize profit over care reflecting broader skepticism [22].

Rather than contradicting the Health Belief Model (HBM), awareness of Cancer Alley’s high cancer rates illustrates how high perceived susceptibility may coexist with substantial perceived barriers to preventive healthcare-seeking [31]. While participants recognized cancer risks, they primarily sought medical care only after a diagnosis or when symptoms became serious. The HBM identifies perceived barriers alongside susceptibility, severity, benefits, cues to action, and self-efficacy as factors shaping health behavior [32,33]. In this study, financial constraints, transportation difficulties, limited access to specialists, healthcare mistrust, and insufficient information about preventive services may help explain why a strong sense of cancer vulnerability did not consistently translate into preventive action. Therefore, the findings do not indicate a failure of the HBM; rather, they demonstrate the importance of considering perceived susceptibility together with the structural and interpersonal barriers that can constrain action.

Consistent with the Theory of Health-Seeking Behavior (THSB), participants’ accounts also demonstrate that care-seeking was shaped by symptom recognition, previous experiences, available treatment options, and exposure to the healthcare system [35,36]. Financial pressures also formed part of the broader context in which participants made healthcare decisions, consistent with evidence concerning the financial burden of cancer care [21]. Residents must know about early screenings to use them, yet findings show little communication or special campaigns in recent months. This issue became particularly apparent in the contrast between residents who reported little awareness of screening opportunities and the healthcare communication professional who described existing outreach and free screening programs. This discrepancy suggests that the availability of a service does not necessarily ensure community awareness of or engagement with that service. This communication gap, combined with healthcare mistrust, further limits proactive health-seeking behaviors, reinforcing THSB’s links between health actions and sociocultural factors.

Taken together, CMIS, HBM, and THSB provide complementary explanations for the care-seeking patterns identified in the interviews. CMIS helps explain how previous experiences and access to useful information shape healthcare knowledge; HBM helps explain why recognition of cancer risk may not result in preventive action when substantial barriers remain; and THSB helps explain how those information, experiential, and structural conditions influence when and how individuals ultimately seek care. The findings therefore suggest that information seeking, perceptions of risk, and healthcare-seeking behavior should not be understood as isolated processes in an environmentally burdened community.

### 6.1. Recommendation for Preferred Information Channels for Residents

Raising awareness among residents exposed to pollutants is crucial for early detection and reducing mortality. Most participants, except the healthcare communication professional, agreed that the current outreach is insufficient. When asked about preferred methods for receiving healthcare information, participants suggested TV ads, brochures, mailers, billboards, community outreach events, town halls, libraries, and in-person communication at churches and grocery stores. Three emphasized social media, and one mentioned webinars as effective for young people, though two noted digital methods often exclude older individuals. While some preferred brochures in mailboxes, others stressed the need for frequent updates. One participant stated, “The best way to get information is to research yourself,” but acknowledged this is difficult for elderly individuals due to digitization.

John (WM), a journalist, argued that building more healthcare facilities alone will not solve the issue, emphasizing the need to address poverty and cultural attitudes toward healthcare. He stressed that, “People do not have access to healthcare in general. They do not have a doctor. They do not have a gynecologist. They just show up at the hospital, are pregnant, and ready to have a baby.” This highlights the need for an entire re-education of health-seeking to shift people from reactive healthcare behaviors (only seeking care in emergencies) to a more proactive approach that emphasizes prevention and regular checkups.

Romela (AF), another professional who worked in healthcare communication, described how unawareness could create mistrust in the healthcare system and recommended that health communication professionals work on building trust among vulnerable individuals by sharing adequate information they need and reassuring them about the care they will receive. She recommended grassroots communication efforts in local communities, such as in churches, grocery stores, and community events that they might have in that area. A grassroots effort is an initiative that brings together a significant number of people to advocate for subjects that they care about. These people are organized and mobilized at the local level rather than by central leadership [47]. Residents recommended learning opportunities that can help establish trust. They emphasized the importance of having individuals on the ground to come out and speak with people face to face in order to create trust. Another essential suggestion is to raise awareness and facilitate early screening for cancer. Grassroots communication strategies, such as local gatherings, church events, and community health fairs hosted by healthcare organizations, could raise awareness about the necessity of early screening and educate people about the region’s high cancer prevalence. Another technique to assist residents in distributing information is to provide important phone numbers and contacts of essential healthcare services on a proactive basis. If not the government, local legislators should ensure that everyone in the area has a list of clinics and hospitals, complete with phone numbers.

### 6.2. Study Limitations

Several limitations should be considered when interpreting these findings. First, this was a small exploratory qualitative study involving nine participants, seven of whom were Cancer Alley residents. Recruiting residents who were willing to discuss personal experiences related to healthcare, environmental exposure, and community life was challenging, which led to the use of snowball sampling. Recruitment of environmental justice advocates and other professionals was also difficult because of limited availability and willingness to participate.

As a result, five of the seven resident participants were from St. James Parish, and the sample does not capture the full geographic, demographic, or experiential diversity of communities commonly included within Cancer Alley. Although the journalist and healthcare communication professional provided useful contextual perspectives on information dissemination and stakeholder recommendations, the small number of professional participants also limits the breadth of those perspectives.

Another limitation is the reliance on self-reported data, which may be influenced by recall bias or other response-related biases. In addition, the use of Zoom created technological barriers for some older potential participants, which may have affected recruitment and participation. These limitations should be considered when interpreting the findings, which are intended to provide an exploratory account of participants’ experiences rather than represent all residents of Cancer Alley.

Because this study involves a small and geographically bounded community, pseudonyms cannot completely eliminate the possibility that participants may be recognizable to individuals familiar with the local context. This possibility was considered when presenting participant characteristics and quotations.

The predominantly deductive analysis is a limitation because theory-informed coding may have emphasized anticipated concepts while making unexpected interpretations less visible. Although cross-cutting patterns such as the service-awareness gap and the coexistence of high perceived susceptibility with reactive care-seeking emerged across participants, a more inductive or abductive approach might identify additional themes.

### 6.3. Recommendations for Future Research

The researcher recommends further studies on healthcare-seeking behavior among vulnerable populations, particularly in areas with significant environmental health impacts. Exploring healthcare-seeking opportunities and awareness in regions like Cancer Alley is crucial, as is examining trust in healthcare providers and proactive healthcare usage, which emerged as key themes in this study.

The discrepancy identified in this study between healthcare outreach reportedly being available and residents’ limited awareness of those services also warrants direct investigation. Future studies could compare organizational records of outreach and screening programs with community awareness, message exposure, trust in communication channels, and actual service utilization. Such research could help distinguish whether barriers arise from the absence of services, insufficient dissemination, limited message reach, lack of trust, or other accessibility constraints. Longitudinal research should further examine what happens after delayed or reactive care-seeking. Future studies should examine whether delayed or reactive cancer healthcare-seeking translates into greater multidimensional care needs once patients enter the healthcare system, considering clinical, functional, psychosocial, and care-related complexity as complementary dimensions of the cancer experience [48].

## 7. Conclusions

This study highlights that healthcare-seeking behavior and awareness in Cancer Alley are influenced by factors such as distrust in healthcare, financial limitations, transportation issues, and the lack of specialized care. Personal and familial health histories, impacted by industrial pollution and health disparities, also play a role. The study identifies effective communication methods for this underserved community and suggests a multifaceted approach to overcome barriers to proactive healthcare. Residents and professionals emphasized the importance of health education and direct outreach to build trust. Several resident accounts reflected reactive rather than preventive healthcare-seeking, including delaying care until symptoms became serious or reporting limited engagement with preventive screening. To address this, healthcare organizations should focus on trust-building through culturally appropriate messaging and grassroots outreach, using trusted local figures and collaborating with advocacy groups. Two-way communication is critical to address concerns and empower the community to seek healthcare. While healthcare providers should promote services, residents should also be more proactive in seeking care and participating in preventive activities like screenings, which can improve outcomes through early detection.

## Figures and Tables

**Table 1:** List of Interviewed Participants.

Participant	Participant Category	Race/ Ethnicity	Gender	Occupation
Latoya	Resident & Environmental Justice Advocate	Black or African American	Female	Teacher
Marcus	Resident	Black or African American	Male	Chemical Plant Worker
April	Resident	White	Female	Marketing Intern
Tyrone	Resident	Black or African American	Male	Chemical Plant Worker
Lucia	Resident	Hispanic or Latino	Female	Lab Analyst at Chemical Plant
Jasmine	Resident	Black or African American	Female	Education Coordinator
Melisa	Resident	Black or African American	Female	Court Office Clerk
John	Professional (Journalist)	White	Male	Reporter
Romela	Professional (Health Communication Professional)	Asian	Female	Communications and Program Specialist

**Table 2:** List of Findings.

Analytical Theme	Theory/Model	Findings
Access to Care Is Shaped by Intersecting Financial, Geographic, and Trust Barriers	Connected to the literature review, including the environmental and health issues prevalent in this region, structural racism, and the barriers to healthcare.	Participants had varying knowledge of cancer facilities within 100 miles. While most citizens are aware of available cancer care hospitals near their homes, some remain unaware of where to seek cancer care in Louisiana. This information is limited to knowing the names of hospitals and medical centers, but not health service offerings or preventive care. Most participants were open to traveling long distances for quality care (e.g., Texas). Financial constraints and personal experiences with cancer impacted decisions.
Access to Care Is Shaped by Intersecting Financial, Geographic, and Trust Barriers	Comprehensive Model of Information Seeking (CMIS)	Mistrust toward healthcare providers reported by some older Black participants. Low trust in government and mixed trust in local leaders. Financial limitations such as high costs of treatment and medication. High cost of transportation and commuting to the care centers. Lack of local specialists requires travel. Proximity to healthcare and accessibility.
High Perceived Cancer Susceptibility Coexists with Reactive Care-Seeking	Health Belief Model (HBM)	All residents associate cancer risk with location and chemical exposure. All participants feel more likely to develop cancer due to environmental factors. Cancer history among friends and family creates more sense of vulnerability. Concerned about throat, breast, colon, and prostate cancer. Reported complicated health issues: specific mutations, asthma, autoimmune hepatitis, Hashimoto’s thyroiditis, and Hashimoto’s encephalopathy. All participants know individuals affected by cancer. Each resident reported at least one cancer-affected family member. Some noted 4-6 cancer incidents within their families or neighbors. Limited proactive care: Just 1 among 7 engaged in preventive screenings, often discovering cancer during routine exams.
The Service-Awareness Gap: Outreach May Exist Without Reaching Residents	Theory of Health-seeking Behavior (THSB)	Participants exhibited differing degrees of awareness regarding available healthcare services. 8 out of 9 indicated a lack of information about healthcare offerings. Lack of health information and outreach in the community was noted. Lack of two-way communication was noted. One resident: “Healthcare organizations are still communicating about COVID.” A professional noted that outreach efforts primarily occurred during COVID-19. 3 out of 7 residents mentioned local environmental advocates working to raise awareness, e.g., Rise St. James.

## Data Availability

The interview data are not publicly available because they contain potentially identifying contextual information from participants residing or working within a geographically bounded community. De-identified excerpts supporting the findings are presented in the manuscript. Additional data cannot be shared where doing so could compromise the confidentiality protections provided to participants.
